# Mr. Hicks, in Reply to Mr. Ward

**Published:** 1807-09

**Authors:** George Hicks

**Affiliations:** Baldock


### *To the Editors of the Medical and Physical Journal.*

GENTLEMEN,

BEING convinced in my own opinion, that William Miles's Case, which you did me the favour to insert in your Medical and Physical Journal, No. 97, page 272, proceeded from the bite of a dog, and from the symptoms believing it to be genuine hydrophobia, together with the singular way in which he was relieved by the application of a caustic,* I felt it my duty to communicate it to the public; and notwithstanding the very ingenious remarks of Mr. Ward on the same case, in your subsequent Number, I still continue in the same opinion, and should have made an earlier reply, had not the very numerous engagements I have met with, kept me from it till within these few days. From the great haste in which Mr. Ward made those remarks, I am inclined to suppose he has misconceived me, not only in the length of time Wm. Miles took the medicine, it being five days and a half instead of seven, but in many other instances; and was he to reconsider the case, perhaps he might find it less inaccurate than he at first imagined; particularly, if he takes into the account the great variety of appearances which take place in nervous affections, and in none more than in hydrophobia.

The Edinburgh Practice of Physic, vol. 2, page 444, observes, "In this disease the symptoms are so various, that they cannot be enumerated, for we seldom read of two cases of hydrophobia, which do not differ very remarkably in this respect."

Having recorded what past prior to my seeing Miles, and that, as near as I could, in the way it was given me, may make it appear to some, not only inaccurate, but likewise to contain a great deal of extraneous matter; for instance, what passed in his mother's house, and followed immediately after he left it. "And, as she says, his throat projected out almost to his chin, he grated his teeth, and appeared incapable of swallowing his saliva." But had I omitted any part, the case would not have been fairly stated to the public, and I should have been guilty of

* I hope I may be allowed the expression, having never read or heard of an hydrophobic patient being cured by the application of a caustic.

of a much greater fault. The only idea his mother's account gives me, is, that at that time the spasmodic affection of his throat was great, and though she expressed herself in such strong terms, (which is not unusual with the lower order of people,) I have not the least conception the projection was so great as she mentioned, though there must certainly have been a considerable fulness, or it would not have struck her so forcibly. It likewise appears, he then laboured under a strong excitement of the nervous system, from whence his mind became hurried and agitated, which occasioned the hasty reply he made when she asked him to drink some beer, "Drink, no! I have been bit by a mad dog." Had she asked him to have eaten, his reply in all probability would have been, "Eat, no! I have been bit by a mad dog." It seems his principal reason for calling on his mother, was to make her acquainted with his situation, who till then, knew nothing of the bite, he having resided at his master's. At that time, he had not the least aversion to water, though he swallowed with difficulty; at sometimes, more so than at others, as the spasms of the fauces were more or less violent. The spasmodic affection of the fauces was always relieved for the time by the antispasmodic mixture, which accounts for his taking it so regularly: indeed, upon the striking of the clock in the next cottage at the end of every two hours, he would ask for his mixture, which could not be from its being pleasant to his palate, but from the effect it had as a topical application on the fauces, though it did not appear to have the least on the system. He said, upon its being put into his mouth, his throat felt for a short time more open, which enabled him to swallow it with more ease than other fluids. However, the excitement on the nervous system might have acted on a weak imagination,* I cannot suppose the acts Miles committed

* Mr. Ward says, that the symptoms appear to have been much aggravated by the influence of fear or terror upon a weak imagination. Fear or terror, certainly, had not the least influence over Miles prior to the system being affected by the virus generated in the cicatrix, though from the distrest feelings he experienced afterwards, he became much agitated; which agitation, in a great measure, subsided on his being secured, when he became calm and collected in his mind There still, however, remained a disposition to scratch; for on the third day of his confinement, the two men who had the care of him, took off his handcuffs and were in the act of unbinding him, when he began to scratch, and the force with which he struck his small clothes (which were of the thick plush kind) with his nails was mitted to have the least resemblance to those of a maniac who is disposed to commit much greater acts of violence than scratching and biting. It is not uncommon for the maniac to shew the greatest inveteracy against his nearest relatives. Some years back, I met with an instance of a maniac, who making his escape from St. Luke's, the moment he entered his father's house, knocked him down, and had it not been for those present, would have killed him: whereas, the conduct† of Miles to his mother shewed the greatest love and affection towards her. However great Mr. Ward's experience may be, both from practical observation and extensive reading, I cannot agree with him, that "the short space of time (ten days) which intervened between the bite and the accession of the disease, is a circumstance so unprecedented in the history of hydrophobia, as to afford the most satisfactory evidence against the rabid, and in favour of the tetanic origin of the disorder." We are informed in the Edinburgh Practice of Physic, vol. 2, page 443, that, in some few instances, the disease has commenced in seven or eight days from the accident. Motherby says, the symptoms in some take place in two or three days after the bite, more frequently not till after as many weeks. Dr. Hamilton, of Ipswich, is of opinion the disease takes place in a few days after the bite. M. Sabatier in the five hydrophobic cases he gives, in the Medical and Physical Journal, Nos. 97 and 98, (which proved fatal) observes, that the disease appeared at different periods from the time of the injury. The first patient was occasionally attacked with vertigo from the very day on which he received the wound; in a short time, he complained of a weight in his head, and his ideas became confused; his features were much distorted, he past restless nights, and his sleep was continually interrupted by frightful dreams: every symptom

---

was such as to cut holes through them; upon which the men secured him as before. Had he been a subject easily impressed with fear, his fears would have induced him to have applied for a remedy as a preventive. We have heard of some, who, not through fear only, but who have prudently judged it proper to have the bitten part removed by excision or caustic, and others, who (in all probability through fear) not being satisfied with the excision of the part only, have undergone amputation.

† It is not an uncommon thing with those who have the misfortune to be afflicted with this dreadful disease, to give the same caution to their friends, as Miles gave to his mother in the hovel. "Conscious of the approaching inclination to bite, he warns his friends of their danger, and advises them to keep at a distance."—Motherby.

tom indicated approaching disease. Dr. Houlston gives an account of a pamphlet published at Paris by order of Government, in which we are informed, out of fifteen persons bit by a mad wolf, on the 8th and 9th of December, 1775, four died in a few days raving mad; the eleven were put under the care of M. Blaise of Cluny.

One of the eleven, who had, for ten days exactly, followed the plan laid down (as preventive) became melancholy, was seized with a horror and dread of fluids, and died within forty-eight hours afterwards. Nor can I agree with Mr. Ward, that the exposure to cold and moisture* on Sunday, November the 30th, was sufficient, without

---

* Cold and moisture certainly, in some parts of the globe, are not unfrequently the cause of tetanus; and those who are but little acquainted with the disease, from the following observation of Mr. Ward, may be led to suppose, that it frequently takes place in this Island from that cause. "It is well known by every person, who is at all conversant in medicine, that nothing is more common than for tetanus, with all its dreadful train of symptoms, (compared with which, those of Mr. Hicks's patient are scarce worth mentioning) to ensue from a much less degree of exposure to cold, than that which evidently took place in the present instance."

From whatever authority Mr. Ward may have advanced this assertion, I am inclined to think, that those of the most extensive practice, have met with very few tetanic cases originating from this cause. Dr. Lionel Chalmers, in the first volume of the Medical Observations, which is supposed to be equal, if not superior to any thing on the subject that has been offered to the public, says, "Happy it is for the inhabitants of the more temperate climates, that such diseases appear very rarely among them; but, in those countries which lie in the more southern and warmer latitudes, they are endemic, especially to Negro Slaves. In South Carolina they shew themselves at all seasons, but not so often in the winter, more frequently in the spring and autumn, and are most common in the summer, when the people work abroad and are alternately exposed to the scorching heat of the sun, and heavy showers, which often happen suddenly, and greatly alter the temperature of the air. Others are seized with opisthotonos, after sleeping without doors, that they may enjoy the deceitful refreshment of the cold night air when the weather is warm. He farther observes, that these diseases so rarely appear as originals in Europe, that a good description of them cannot be expected from the physicians who practice in that part of the world; nor, says he, has any thing like a full description of them been given by any ancient or modern author which I have seen. Hippocrates, indeed, takes notice of them in many places, and seems to regard them only as consequences of other diseases, or of wounds or ulcers of the nervous or tendinous parts."

Should Mr. Ward have met with a sufficient number of tetanic cases, originating from cold and moisture only, and that in the winter, to confirm him in his opinion, that Miles's indisposition originated from that cause, those cases, without doubt, would be very interesting to the public; and as he has a sincere wish to increase to the utmost of his power, the resources without supposing the dog to have been mad, to account for all (and much more than all) that followed. Whatever opinion Mr. Ward may entertain of Miles's case subsequent to Sunday, November the 30th, he most certainly must agree with me, that cold received on that day, could not produce the stiffness of the knee and thigh, which took place on the Saturday preceding, nor could it occasion the morbid irritability of the skin, which came on, on the Saturday night, nor the stiffness of his throat, and difficulty of swallowing, he experienced on the Sunday morning in eating his breakfast.

If Mr. Ward really takes the case to be tetanic, he might with much greater propriety suppose the tooth of the dog to have punctured a nerve, and that the system became affected thereby, and not by cold and moisture. From what I have seen of tetanus, I readily allow, that the puncture of a nerve by the tooth of a dog may bring it on; but it appears to me that if Miles's indisposition had originated from the puncture of a nerve only, and not from virus, the symptoms would have been different. In tetanus there are three varieties, which are classed according to the muscles that are first affected, and the posture into which the body is thrown, from the contraction of those muscles. The variety which is most common, begins with the contraction of the muscles of the back part of the neck, shoulders, jaws, and fauces, with pain of the lower end of the sternum; from the shoulders the contraction extends down the spine and bends the trunk backwards, from whence it is called opisthotonos. Then the muscles of the lower extremities become affected in the same way; and, lastly, the anterior muscles become equally contracted with the posterior, so that the whole body is rendered inflexible. When the contraction of the flexors of the head and trunk is equal to the contraction of the extensors, so as to make the body straight, and incapable of being bent any way, it is called tetanus: but should the contraction of the flexor muscles not act equally against the extensors, the spine will remain incurvated, and an arch will be formed from the heels to the head; in this

---

resources of medicine and surgery, I flatter myself we shall soon be favoured with them.

To me it appears more likely, that Miles's indisposition proceeded from virus generated in the cicatrix, and that the action which generated the virus, was excited by the saliva of the dog introduced into the wound, or rather by the virus contained in the saliva.

this case it retains the name of opisthotonos. And when the flexor muscles of the head and trunk are first contracted, it is called emprosthotonos: This variety very rarely occurs; Dr. Cullen has omitted it, and added trismus, which appears to be a slight degree of opisthotonos, or, (where the disease goes on) an incipient tetanus.

In hydrophobia, the fauces, œsophagus* heart,† trachea, intercostal muscles, diaphragm, abdominal muscles, stomach, intestines, &c. are the parts affected with spasm; from whence we may conclude, that the parvagum and intercostal nerves, are the nerves principally affected, whereas in tetanus, the system becomes generally affected;

Hydrophobic patients have a disposition to bite, which, as far as I have seen, does not take place in tetanus.

The dread of water in tetanus appears to be produced in a different way to that which takes place in hydrophobia. In tetanus it originates in spasmodic affection of the muscles subservient to deglutition being increased on an attempt to swallow, which is attended with great pain, and increases the spasmodic affection of the whole system, the smallest quantity passes with the greatest difficulty into the stomach, the stricture being, from one end of the œsophagus to the other; whereas in hydrophobia, the sight of water, of a glass, or of any thing pellucid, will bring on horror immediately, with an increase of the spasmodic affection; and if the patient by dint of resolution can get any thing to pass the fauces into the œsophagus, it passes very readily into the stomach.

On whatever part of the body the wound has been inflicted, the hydrophobic virus seems to have a specific action on the parvagum and intercostal nerves; and from the nervous communication there is between the intercostal nerves, and the ophthalmic branch of the fifth pair of nerves and from thence to the retina, may arise that peculiar morbid irritability of the retina, which occasions terror to take place on the appearance of water, or any thing pellucid: as likewise, from the same nervous com-

 munication,

---

* At the commencement of the disease, it appears, that the œsophagus is not much affected with spasm, (though it becomes so afterwards,) from fluids, after they have passed the fauces, having a ready transit into the stomach.

† The pulse continuing natural, like a sound healthy pulse, for sometime after the attack of the disease, shews, that the heart is not affected, till towards the close, when the pulse becomes quick, weak, and intermittent.

munication, may proceed the spasmodic affection of the fauces, &c. which becomes visibly increased on the appearance of water. Strangulation of the throat is felt, and the larynx becomes outwardly swollen.*

Why Mr. Ward should suppose the recurrent nerves to be the principal agents employed in producing the characteristic symptom of hydrophobia, a dread of water, I confess I cannot make out. The recurrent nerves are given off from the parvagum just as it enters the thorax, the right near the axillary artery, the left a little lower; they both ascend and are lost in the muscles of the larynx and pharynx. It is a known fact, in experiments on dogs, that on cutting the intercostal nerves, their eyes become dull, they lose their bright water, and have a large secretion of gum or gore, the pupils become contracted, the eye balls diminished, and the cartilaginous membrane at the internal canthus comes more over their eyes.

Near sixteen years ago I was called to James Tyler, a pauper of the parish of Weston, about 13 years of age, who a few weeks before, as he was bringing a ploughshare out of the field on his shoulder, with the palm of his left hand applied to the point, had the misfortune to kick his foot against the root of a tree, which threw him down: his left hand coming to the ground in this position, the point ran into the palm of his hand, and produced a wound, which, considering the manner in which it was inflicted, was but small. It was, however, in great pain for two or three days, during which time his mother poulticed it: she afterwards dressed it with cerate, and it appeared to be well. In consequence of which, the preceding evening to my being called to him, he applied to his master for work, and being informed he might come to work again the next morning, he returned home in high spirits. After eating his supper, he was taken suddenly, as his mother supposed, in a fit; he fell backward out of the chair on the ground; upon taking him up, his jaws were locked, his neck and shoulders stiff, and his head drawn backward, as in the first stage of opisthotonos. She still, however, supposed it was a fit, and all that was given him that night was a few drops of hartshorn in water, which was got into his mouth, through an opening in his teeth,

* This was strongly marked in Miles, for on water being brought to him on Friday night, when at the distance of two or three feet, his throat became much affected, and, as Mr. Baron, of Walkern, who was present observed, it was much enlarged.

teeth, (he having lost a front tooth) which he did not swallow. In the morning, his mother applied to the parish for medical assistance; I was sent for, and found him in this state: no part of his body would bend in the least, he had a small, fluttering pulse, with cold perspiration. He expired soon afterwards.

I was called to William Honour, of Snailsworth, on Thursday, October 29, 1801, and found him in a dying state from a tetanic affection. On inquiry, I was informed he had pricked his right leg with a spur on the Monday evening preceding, October 26; the puncture was very small, a mere speck, about four inches above the inner ankle; it appeared but little inflamed, which unfortunately led Honour and his friends to suppose no harm would ensue, and kept them from applying for assistance till it was too late.

January 23, 1802.—I was called in the evening to a blacksmith of this town, who, in shoeing an unruly horse about noon the same day, pricked his leg with a nail; it gave him great pain at the time, which was now become very violent; he complained of pain under the sternum, with some difficulty of breathing. I immediately applied a saturnine poultice to the part, with a large quantity of tincture of opium in it, and gave him a large dose of opium internally every four hours. In the morning I had the satisfaction to find the pain very much abated, and the spasmodic affection entirely removed. The same application was continued to his leg for that day, and on the following he was perfectly free from pain. In this case, had not the patient been relieved so soon by the opium, I have every reason to believe, a caustic applied to the part would have produced a happy effect. Some other tetanic cases I could mention, which have occurred in my practice, proceeding from punctures; one very lately in a respectable farmer, who in putting on his coat was pricked in the arm by a needle, which was left in the lining, (the lining having been lately mended.) As soon as he felt the needle prick him, he pulled off his coat; the needle came out at the same time; the puncture was scarcely visible. The next day, his arm was stiff and painful; the stiffness extended up his shoulder and neck, towards his jaw and cheek on that side. In this situation he sent for me; I gave him an embrocation consisting of tincture of opium, with a large quantity of camphor dissolved in it, and one fourth part of Spt. Ammon. C. directing him to embrocate the part affected with it, very often, and gave him a large dose of opium every four hours; at the same time informing him, if he was not soon relieved, it would be necessary to apply a caustic to the part. By these means, however, I had the satisfaction to find the pain and stiffness soon removed.

Miles was bit on the 19th of November; the bite was very small, a mere scratch with a single tooth; it healed up apparently well, and continued to look well till Thursday, November 27, when it itched and appeared rather red and elevated. On Fridy the 28th, the redness increased, with slight prickling pains in it. Saturday the 29th, the prickling pains* increased; about noon his knee became stiff, which stiffness, in the course of the afternoon, extended up the inner part of the thigh; in the evening he was taken with coldness after he was in bed, very different to rigor proceeding from absorption; no increased heat followed, nor was the pulse increased in its action; † there appeared to be a morbid irritability of the skin, in consequence of which, it could not bear the stimulus of the external air, which occasioned him to cover himself over with the bed clothes, saying, he could not bear the cold wind to come to his face. Nov. 30, upon getting out of bed in the morning, the sensation of coldness increasing, he went to the fire; and in eating his breakfast, found his throat stiff, and swallowed with difficulty; soon after which he felt himself much agitated, and could not keep himself still, in consequence of which he went into the field: ‡ after some time, a sudden depression of muscular strength coming on, he fell down;§ and upon getting up, was

---

* Motherby says, "the disease comes on with slight pains in the wound, sometimes attended with itching, but always resembling a rheumatic pain; it extends also into the neighbouring parts, and at length from the extremities it passes into the viscera. This pain is considered as the primary invariable mark of a beginning hydrophobia."

The Edinburgh Practice of Physic, vol. 2nd, page 443, in giving the description of hydrophobia, observes, "the approach of the disease is known by the cicatrix of the wound becoming high, hard, and elevated, and by a peculiar sense of prickling in the part, pains shooting from it towards the throat."

† Edinburgh Practice of Physic, vol. 2nd, page 444. "Some complain of the coldness of the air frequently, when it is really warm."—Motherby. "In some an exquisite sensibility is induced, so that the air offends if it touches them."

‡ During the whole of his indisposition, he had a strong desire to go into the field, as if he wished to be solitary.

§ It does not appear to me he layed long on the ground. I attribute his falling was taken with pain* and stiffness at the pit of his stomach, which extended up to his throat, as if he had been bound with strong cords. He now became more hurried, and knew not where he went, having, from his distressing feelings, a very imperfect idea of what past, till after he was secured, when his reason returned. He had a strong disposition to scratch and bite, as appears from his biting the two men, &c. Dec. 1st, 2nd, and 3rd, he continued much the same, excepting the spasmodic affection of the præcordia and fauces being gradually increased. On Friday, Dec. 5, I called upon him in the evening, and was informed by his attendants, that on the preceding day, Dec. 4, he became much agitated at the sight of water, and would not suffer it to approach his lips, saying "it terrified him," and that the spasmodic affection of his throat became much increased upon its being brought to him. During the whole of the time from the Thursday evening, the only fluid he swallowed, excepting his medicine, was a spoonful or two at most, of broth, with the greatest difficulty, and that at a great many times, though he made use of every effort. They said, he appeared very fond of apples,† and requested to have slices put into his mouth, which they frequently did, and he swallowed them with ease. I then asked for a cup of water, which was brought, and as soon as he saw it the spasmodic affection was visibly increased, so that it was noticed by those who were present; and he expressed himself much terrified.

I had him raised up upon his breech, and it was with the greatest difficulty the two men and myself, with the assistance of another person, could get the water to his mouth. Upon forcing some of it into his mouth, entreating him at the same time to swallow it, he could not; it returned again. From these appearances, I have every reason to conclude that Miles's indisposition was genuine hydrophobia, and that if he had not been relieved by the caustic

---

falling to an increased nervous affection which took place at that time, and produced a sudden depression of muscular strength, which was only momentary, or continued but a short time; that he then experienced an increased nervous affection, is evident from the spasmodic affection of the præcordia, &c. which immediately followed.

* "The oppression of the præcordia is one of the primary and constant symptoms of this disease; it begins, increases, and ends only with it."—Motherby.

† Edinburgh Practice of Physic, vol. 2nd, page 444. "Sometimes they can swallow bread soaked in liquids, slices of oranges, or other fruits."

caustic that was applied that evening, he would have died; and though he had none of that spitting of saliva, which is very common towards the close of the disease, I believe it would have taken place, as there appeared to be a greater quantity of it about the fauces, on the Friday evening, than I had observed before.

I have been favoured with the following account of John Gillet, of this town, who died of hydrophobia on the 8th of May, 1794, from the bite of a mad dog about Christmas preceding, from the minutes that were taken by the gentleman who attended him. He was attacked on the 5th of May in the evening with pain about the præcordia, past a restless night, and on the following morning, May the 6th, was taken with a spasmodic affection of the fauces, and could not eat his breakfast, which was followed with a difficulty of breathing and a dread of water. These symptoms increased till he expired. Upon the appearance of a glass, or any thing pellucid, the spasmodic affection of the fauces, &c. was increased to a great degree, as well as the terror and distress he had about him. On the 6th and 7th, he took a few doses of medicine, which he swallowed with difficulty; he continued sensible till within two or three hours of his death, when delirium came on. From the account of his sister, who constantly attended him, he had not that flow of saliva which is common, and therefore not that spitting. He requested to have slices of apple given him, which he swallowed.

As the following case of Thomas Surry tends to shew the utility of excision and caustic, as preventive of hydrophobia, I have thought proper to annex it.

About the middle of July, 1799, a mad dog, in passing through Baldock, bit three or four dogs belonging to a gentleman of this town. The dogs were confined in the kennel. About ten or twelve days after, Thomas Surry, whose employment it was to look after the dogs, observing one of them look heavy and dull, with its eyes gummed up, very imprudently, (knowing the dog to have been bit by a mad dog) sponged them with water; in doing of which, the dog bit him on the second bone of the left thumb, and produced two lacerated wounds of the integuments, not sufficiently deep to injure the periostæum. Fortunately, one of the wounds was on the side next to the fore-finger; and the other, on the side opposite, so that the tendons of the flexor and extensor muscles escaped uninjured.

He

He chained the dog up, after which it refused to drink, and died much convulsed the following day, with its mouth full of frothy saliva. Soon after the bite, Surry applied to me; I removed the part by excision, and lest there should be any remains of virus, I applied a slight caustic to each wound. No other remedy was made use of. He has never had the least unpleasant symptom, and is at this time in good health.

However Mr. Ward may differ with me in many respects, I am fortunate in his agreeing with me, that the caustic had the principal share in effecting the cure, tho' at the same time, he begs leave to differ with me as to the modus operandi. He thinks the good effects of the caustic are to be attributed to its rendering the nerve or nerves situated immediately above, incapable of transmitting what he calls in hydrophobia, the hydrophobic aura to the muscles affected with spasm situated in the superior parts of the body, and recommends the caustic to be applied at the distance of from one to three or more inches above the bitten part, with the view of intercepting the aura in its passage; paying no attention to the necessity there is of putting a stop to the action that is going on in the cicatrix, which generates the aura. Allowing Mr. Ward's hypothesis to be correct, with regard to the generation of the aura in the cicatrix, and that it is transmitted up the nerves to the muscles affected with spasm, it appears to me, that though the nerves above the cicatrix are divided by the caustic in the manner he recommends, so long as the source is suffered to remain, the aura may be communicated by some collateral branch. But till the action of the nervous system is better understood, that is, the manner in which the nerves communicate their influence to the muscular fibres, whatever is offered in this way must appear to me to be mere conjecture. The action of the muscular fibres most certainly depends on the nervous energy; and when that goes on undisturbed, there is harmony throughout the whole muscular system; but when from any cause that is interrupted, a new train of action takes place, corresponding with the cause that produced it, and which may very properly be called the specific action of that cause. It appears to me, the caustic would have a much better effect applied to the cicatrix, as it will not only produce a solution of the extremities of the nerves acted upon by the virus, but decompose the virus, and arrest the morbid action altogether, by which means the nervous energy will will be restored, and the spasmodic affection produced by such morbid action, cease, as in Miles's * case.

I am, &c.

GEORGE HICKS.

*Baldock, Aug.* 10, 1807.

* We have inserted this communication of Mr. H. before its turn, on account of the calm and gentleman-like manner in which he combats the objections of his opponent. When writers have a weak cause to defend, they naturally endeavour to turn the attention of their readers from the real question at issue, by the introduction of collateral matter, by sneers, personalities, or rash assertions. M. H. on the contrary, supports his opinion, solely, by facts and quotations from the most respectable authors that he had an opportunity of consulting.—Ed.

